# Understanding Volume Kinetics: The Role of Pharmacokinetic Modeling and Analysis in Fluid Therapy

**DOI:** 10.3389/fvets.2020.587106

**Published:** 2020-11-20

**Authors:** Xiu Ting Yiew, Shane W. Bateman, Robert G. Hahn, Alexa M. E. Bersenas, William W. Muir

**Affiliations:** ^1^Department of Clinical Studies, Ontario Veterinary College, University of Guelph, Guelph, ON, Canada; ^2^Research Unit, Södertälje Hospital, Södertälje, Sweden; ^3^Karolinska Institutet, Danderyds Hospital (KIDS), Stockholm, Sweden; ^4^College of Veterinary Medicine, Lincoln Memorial University, Harrogate, TN, United States

**Keywords:** fluid therapy, pharmacokinetics, volume kinetics, hemoglobin dilution, urine output, distribution, elimination, half-life

## Abstract

Fluid therapy is a rapidly evolving yet imprecise clinical practice based upon broad assumptions, species-to-species extrapolations, obsolete experimental evidence, and individual preferences. Although widely recognized as a mainstay therapy in human and veterinary medicine, fluid therapy is not always benign and can cause significant harm through fluid overload, which increases patient morbidity and mortality. As with other pharmaceutical substances, fluids exert physiological effects when introduced into the body and therefore should be considered as “drugs.” In human medicine, an innovative adaptation of pharmacokinetic analysis for intravenous fluids known as volume kinetics using serial hemoglobin dilution and urine output has been developed, refined, and investigated extensively for over two decades. Intravenous fluids can now be studied like pharmaceutical drugs, leading to improved understanding of their distribution, elimination, volume effect, efficacy, and half-life (duration of effect) under various physiologic conditions, making evidence-based approaches to fluid therapy possible. This review article introduces the basic concepts of volume kinetics, its current use in human and animal research, as well as its potential and limitations as a research tool for fluid therapy research in veterinary medicine. With limited evidence to support our current fluid administration practices in veterinary medicine, a greater understanding of volume kinetics and body water physiology in veterinary species would ideally provide some evidence-based support for safer and more effective intravenous fluid prescriptions in veterinary patients.

## Introduction

Fluid therapy paradigms are constantly changing (1–3) due to new discoveries (4, 5) and ongoing debate on the ideal fluid choice, dose, rate, and efficacy in different patient populations (6–8). The limited and poor quality scientific literature on IV fluid therapy in veterinary medicine (9), particularly in the feline species, has given rise to empirical fluid therapy recommendations (10, 11) that are based on broad assumptions, outdated physiological principles, clinician's anecdotal experiences, or extrapolation from human clinical trials and canine experimental models. Extrapolation of data from human and canine studies are not ideal as there may be critical species variation and different fluid kinetics associated with various IV solutions. As a routine treatment of hospitalized patients, fluid therapy has the potential to cause fluid overload which increases morbidity and mortality (12), especially in cats (13), yet we have little evidence to support our current fluid administration practices in this species (9).

The recent discovery by the “Fluid Expansion as Supportive Therapy” (FEAST) study in African children whereby standard fluid boluses significantly increased 48 h mortality (14) took the medical profession by surprise and subsequently raised many questions regarding our understanding of fluid pharmacokinetics. Since then, clinicians are more receptive to the idea that fluids should be considered “drugs” and prescribed according to the four D's (drug, dosing, duration, de-escalation) and four phases (resuscitative, optimization, stabilization, evacuation) of fluid therapy (2, 3, 15). The FEAST study is an important reminder that IV fluid therapy can exert varying physiologic effects dependent upon the context in which they are administered (16) and can be detrimental if administered inappropriately (6, 7).

For decades, research in fluid therapy was constrained by the lack of effective methods to assess important effects and outcomes. Fluid dynamics and volume expansion effects have been investigated using conventional indicator dilution techniques (17, 18), blood volume monitoring (18–20), hemoglobin dilution (21), and bioelectrical impedance analysis (22–24). In 1997, several authors (25–28) pioneered a novel and innovative pharmacokinetic (PK) model adapted for body fluid spaces that could provide descriptive data on the physiologic behavior of infused IV fluids in terms comparable to those employed in conventional PK practice (29).

For the past 20 years, Robert G. Hahn and his research group have been studying and refining the concept of volume kinetics (VK), providing a research platform to understand how administration of various IV fluids affect body fluid compartments under different physiologic conditions. Intravenous fluids can now be studied like pharmaceutical drugs, leading to improved understanding of their time-volume effects or elimination half-life on plasma and interstitial fluid compartments (30), making evidence-based approaches to fluid therapy possible. Although this concept originally served as a research model for humans (30–33), a handful of experimental studies have been performed in animals including rabbits (34), pigs (35), and sheep (36–45).

The study of VK in companion animal species is unprecedented, and there is no scientific literature currently available. In response to the call for more scientific research to build an evidence-based foundation for veterinary fluid therapy, the purpose of this review is to introduce the basic concepts of VK, its current use in human and animal research, as well as its potential and limitations as a research tool for fluid therapy research in veterinary medicine.

## Basic Pharmacokinetic Concepts and Models

Pharmacokinetics and pharmacodynamics are complex disciplines that utilize advanced mathematical principles that practitioners sometimes find challenging to comprehend. However, these two branches of pharmacology are pivotal in understanding ways to enhance efficacy and decrease harm for any exogenous substance prescribed (46), thus critical to clinical drug delivery. Pharmacokinetic analysis and simulation is the established approach to guide pharmaceutical dosing and frequency since it outlines a substance's concentration time course within the body (46). Pharmacodynamics, on the other hand, illustrate the dose-related effect of a substance in the body over time (46).

Absorption, distribution, metabolism, and excretion form the classic principles which make up the “ADME” acronym that describes PK (47). Absorption is the process where a substance travels from the site of administration to the site of measurement (46), while bioavailability refers to the degree and rate that an active substance is absorbed and present at the site of action (48). During and after the absorption phase, distribution occurs when the substance travels to and from the site of measurement (46). Volume of distribution (*V*_*d*_) is a proportionality constant between measured plasma concentration and the corresponding amount of substance within the body according to the state of substance disposition, i.e., at initial time 0 (*V*_*d*(*c*)_), under steady-state conditions (*V*_*d*(*ss*)_), pseudo-equilibrium conditions (*V*_*d*(*area*)_), or at any given time (*V*_*d*(*t*)_) (49). Volume of distribution is primarily designed to determine the appropriate loading dose for rapid achievement of targeted therapeutic plasma concentration, but often interpreted indirectly to encompass the extent of substance distribution following recourse to physiological models involving plasma protein and tissue drug binding (49). Finally, elimination is the sum of metabolism and excretion (46). The term excretion, defined as the irreversible loss of chemically unchanged compound from the body, is routinely confused and used interchangeably with the term elimination, which is the irreversible loss of substance from the site of measurement (50). Most substances are eliminated by a first-order process and occurs when the amount of substance eliminated at any time is directly proportional to the amount of substance in the body, such that the fraction of elimination over time remains constant (51). Zero-order elimination, on the other hand, occurs when the amount of substance eliminated for each time interval is constant regardless of the amount of substance in the body (51).

The management of a substance by the body is an intricate process as the various components of “ADME” occur simultaneously, thereby constantly altering substance concentration in tissues and fluids (51). Application of mathematical principles simplify and help to describe these complex body processes and facilitate the anticipation of a substance's behavior within the body (51). The most fundamental model utilized in PK is the compartmental model, often designated by the number of compartments needed to depict the substance's behavior in the body, e.g., one-compartment, two-compartment, and multi-compartment models (51). The compartmental model is known as a mammillary or catenary model and is dependent on whether peripheral compartments are linked in parallel or in series to the central compartment, respectively (52). In a mammillary compartmental model, the compartments do not represent a specific organ or fluid space, but rather a group of body tissues or fluid spaces that possess similar substance distribution patterns. Blood (plasma) and highly perfused tissues such as the heart, lungs, liver, and kidneys are often collectively regarded as the central compartment (i.e., vessel rich group), while moderately (i.e., muscle) and poorly perfused tissues (i.e., adipose tissue, cerebrospinal fluid) are considered the peripheral compartment (53).

Compartmental models are said to be deterministic because the model that best describes the PK of a substance is determined by the substance's concentrations in the blood (51). If a substance's plasma concentration-time profile best fits a one-compartment model, this denotes that the substance is dispersed instantaneously and rapidly throughout its volume of distribution (53). The plasma concentration-time profile displays a mono-exponential decline as only a single process, i.e., elimination, contributes to the decrease in plasma concentration, while the distribution phase occurs too rapidly to be charted (53, 54). If a substance's PK pattern is better fitted by a bi-exponential model, this suggests that the substance first distributes in the central compartment and then more slowly to a peripheral compartment (53). The substance distributes back and forth between these two compartments and leaves the body from the central compartment (53). This leads to a plasma-concentration time profile with a bi-exponential decline characterized by two distinct phases: (1) the distribution phase which is the initial steep decline in plasma concentration, and (2) the elimination phase which is the subsequent slower decline in plasma concentration sustained by redistribution of substance from the peripheral compartment (54).

The merit of any model depends on how well it mimics the substance's concentration in the body. Typically, the simplest model that sufficiently predicts changes in substance concentration over time is selected (51). A concentration-time curve can be plotted following serial substance plasma concentration measurement and logarithmically transformed to demonstrate a mono-phasic or bi-phasic time profile (53). Subsequently, any unknown concentrations can be predicted and simulated (51). In PK studies, appropriate doses and dosing intervals are determined using computer simulation after repeated experimental measurements of plasma drug concentration are conducted and compared with theoretical computer-generated concentration-time data using non-linear least squares regression (55).

The conventional way to present PK output is to analyze each subject individually and report the mean parameter values for a group as the “typical parameter values” for that cohort. An alternative approach is to analyze all subjects in one single analysis while keeping track of the between-individual variability. The widespread adoption of such population modeling in human pharmacology, also known as non-linear mixed effects (NLME) modeling, provides a framework to characterize sources of variability in drug disposition and response using statistical models that account for between- and within- individual and experimental variation (56–58). Identification and quantification of primary determinants of between-individual variability allow for improved and tailored use of therapeutic drugs, including individualization of dosage regimes and optimization of dosing schedules (58). Population models are referred to as NLME models due to the inclusion of fixed (i.e., constant within the population) and random (i.e., unexplained and varied within the population) effects parameters that possess a non-linear relationship (58). Between-individual variability can be explained by population characteristics or covariates (e.g., species, breed, age, sex, body weight, disease status, etc.) that are included additively or proportionally to the vector of population parameters or fixed effects (58). Random effect parameters represent variability in PK parameter estimates secondary to between- and within- individual variability (58). NLME modeling can be used for analysis of sparse data collected in scenarios where frequent blood sampling is challenging, dense data collected from a small study population, separate plasma and tissue PK studies, and meta-analysis of published data across disparate study designs (57, 58). These attractive advantages of NLME are recognized to be of value with potential widespread applications in veterinary pharmacology (57, 58).

## Basic Concepts of Volume Kinetics

**Figure 1:**
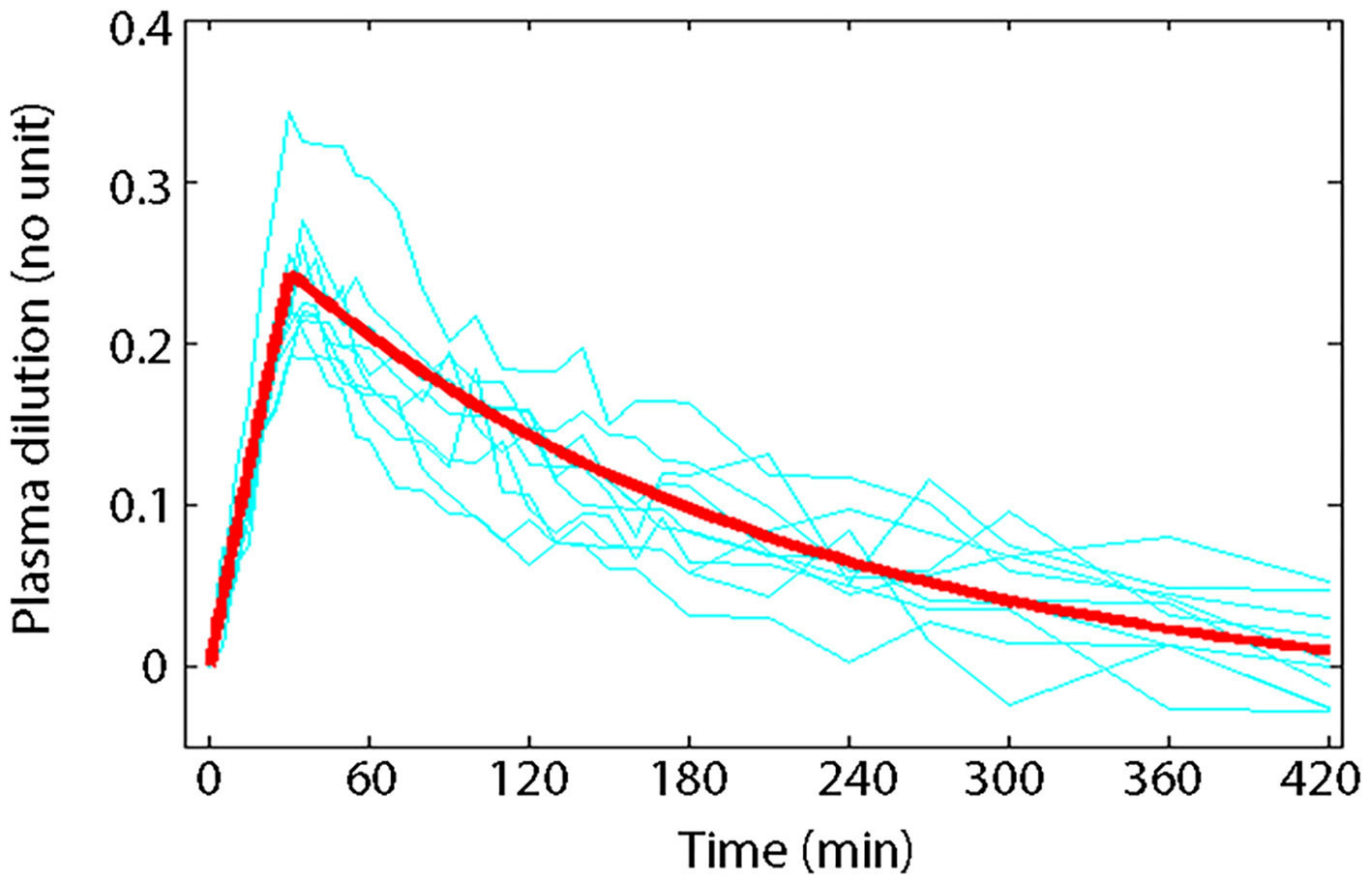
Principle of volume kinetics. Measured plasma dilution-time curves of individual subjects (thin lines) and VK modeled plasma dilution-time curve (thick line) during and after a 30 min IV infusion of 10 mL/kg of 6% HES 130/0.4/9:1 (Voluven®) in 10 healthy male volunteers with mean body weight of 79 kg (59). Serial Hb measurements were obtained throughout the study; hemodilution was corrected to baseline HCT to express plasma dilution. Dividing the total infused volume (790 mL) by the plasma dilution extrapolated to 0.27 at time 0 yielded a *V* of 2.9 L, which approximates the expected size of PV in healthy humans, implying that Voluven® only distributes in the plasma space in health. Plotting the plasma dilution-time curve on a logarithmic paper suggested that Voluven® intravascular half-life is 120 min in healthy humans.

Volume kinetics (VK) is an adaptation of PK theory allowing insights into the effects of IV fluid on theoretical body fluid compartments (25, 30–33, 55). This approach describes changes in plasma volume (PV) during and after IV fluid infusion over time (27). Volume kinetics can also be used to explore the distribution-elimination pattern of IV fluids under various physiological conditions and simulate any infusion fluid choice or rate to achieve desirable PV expansion (32, 33). Visual inspection of the plasma dilution-time curves provides imperative information on the magnitude and time course of PV expansion following actual IV fluid infusion (26). Meanwhile, computer simulation of plasma dilution-time profiles for any hypothetical fluid infusion using the generated kinetic parameter estimates allows insight into how IV fluid therapy should be planned. Figure 1 illustrates an example of information attainable from visual inspection of plasma dilution-time curves (59).

When “ADME” concepts are put into the context of IV fluid administration, the absorption is instantaneous with a 100% bioavailability, metabolism is irrelevant, therefore leaving only distribution and elimination to be considered. The central concepts of both PK and VK analyses are therefore dependent upon the volume of distribution and clearance of an administered substance (31). As in PK, a theoretical mathematical model that corresponds reasonably well to the physiological dynamics of IV fluid is constructed (32, 33). The basic mathematical models developed in VK are known as the volume of fluid space (VOFS) kinetic models. These are designated by the number of fluid spaces necessary that mimic the kinetics of IV fluid within the body akin to traditional PK analysis, e.g., one-volume fluid space (1-VOFS), two-volume fluid space (2-VOFS) kinetic models, etc. (25). Both PK and VK models are admittedly challenging to conceptualize, given that they are mathematical derivatives that do not possess precise tangible anatomical or physiological connections (31).

Unlike pharmaceuticals, water is the main component of both infusion fluids and plasma (30, 31), therefore plasma concentration of IV fluids has to be expressed differently. Blood hemoglobin (Hb) has become an attractive endogenous dilution indicator for blood volume (BV) estimation (21) and correlates inversely with blood water concentration (26). Therefore, serial measurement of acute changes in Hb concentration, which is confined to the intravascular space, can be used as a surrogate marker for IV fluid concentration in VK (32, 55). Serum albumin has been explored as a potential endogenous dilution indicator for VK analysis, however possible loss of albumin into the interstitium negated its use (26). The main assumption of VK modeling and analysis is that Hb remains constant and distributes homogenously with the infused fluid in the circulation, such that serial Hb changes are reflective of IV fluid kinetics.

Volume of distribution (*V*_*d*_) in conventional PK analysis is not easily applied to fluid therapy. Unlike conventional PK, where *V*_*d*_ is assumed constant as the volume of administered drug is often negligible, the volume of distribution of IV fluid (denoted by the symbol *v*) continually changes as a direct consequence of IV fluid administration. Therefore, the baseline output parameter, volume of expandable body fluid space (*V*), representing the volume of distribution of initial fluid infusion is estimated instead in VK (34). Depending on the kinetic model used, *V* can be further categorized as the volume of expandable central body fluid space (*V*_*c*_) and the volume of expandable peripheral body fluid space (*V*_*p*_). *V* can only be estimated from plasma dilution measurement and represents the functional fluid space actually expanded by IV fluid (26). Therefore, *V* may not always equate to the size of body fluid spaces estimated by external indicator-dilution techniques that disperse into the extracellular fluid spaces of minimally expandable structures such as bone, cartilage, dense connective tissue, and organs confined by tight fibrous capsules (34).

Aside from the above interpretation differences, the mathematical solutions and data analysis are analogous to PK compartmental modeling. Volume kinetic models are based on the assumption that plasma dilution follows a mono-exponential (1-VOFS kinetic model) or bi-exponential (2-VOFS kinetic model) curve. The dilution-time profiles, sometimes augmented by urinary excretion (60), are entered into commercial computer software and fitted to the differential equations describing the fluid shifts in these kinetic models (25). Non-linear least-squares regression is used to generate the best kinetic parameter estimates from the differential equations relevant to each kinetic model (25). Subsequently, analytical or matrix solutions to the differential equations are solved to compute *V* as well as the kinetic constants governing fluid distribution (25). Similar to PK modeling, the kinetic model with the simplest solution should be chosen (31) and statistical tests can be applied to select the best fitted model for presentation (61, 62). Agreement between model-predicted renal clearance (*Cl*_*R*_) and actual urine output measurement may also assist in final model selection (32).

Following that, computer simulation of dilution-time profiles for hypothetical fluid infusion can be accomplished by inputting the mean parameter estimates obtained from non-linear least-squares regression into the differential equation solution describing the kinetic model (25). Various commercial mathematical programs such as MATLAB® (MathWorks® Inc, Natick, MA, USA) (63, 64) or specialized PK modeling and simulation software such as Monolix (Lixoft, Antony, France), Phoenix WinNonlin® or Phoenix® NLME^TM^ (Certara USA, Inc., Princeton, NJ, USA) (65) can be utilized for this purpose.

## History and Evolution of Volume Kinetic Modeling

The original VK models were first developed in 1997 based on the concept of clearance and plasma dilution (25–27). The most commonly encountered problems with earlier VK analyses included overestimation and poor precision when estimating the size of the peripheral fluid space (*V*_*p*_), which became an issue with slow fluid elimination (25, 26, 66, 67). Over the years, efforts to refine and improve the robustness of these models were made, including the incorporation of urine output as an input variable to VK analysis (60).

Parameter estimates from earlier VK studies are challenging to interpret and compare because they are derived using different kinetic methodologies. This eventually led to a major modification to the VK approach in 2006 which allows for a single comprehensive model solution for isotonic crystalloid solutions (67). In this modified “micro-constant” analysis, the fractional plasma dilution is substituted with absolute volume expansion, flow rate (unit: mL/min) parameters are substituted with rate parameters (unit: /min), and the existence of *V*_*p*_ is acknowledged but its size is not routinely estimated (67). Despite these changes, this modified model still describes the same functional system as the antecedent models (27, 60, 66, 68) and is able to address bi-directional fluid flux, i.e., outflow when plasma dilution is high, inflow when distributed fluid exerts greater force than *V*_*c*_ (67). Since then, progressively sophisticated extensions have been explored to address specific clinical research questions including three-volume kinetic modeling which accounts for hypertonic osmotic fluid shifts (37, 68), volume turnover kinetics to predict fluid dynamics following hemorrhage and fluid resuscitation (41), and population kinetic modeling that enables the inclusion of various physiological covariates (69–74).

Over the past 10 years, VK has gradually moved away from the clearance model to the micro-constant model where clearance variables (*Cl*, *Cl*_*d*_, *Cl*_0_) are substituted by rate constants (*k*_10_, *k*_12_, *k*_21_, *k*_0_) and fractional plasma dilution ($\frac{\left(v_{c}-V_{c}\right)}{V_{c}}$) is replaced by absolute volume expansion (*v*_*c*_ − *V*_*c*_) (32, 33) in the kinetic equations. The clearance and micro-constant models are essentially one and the same, except that the former deals with fractional plasma dilution and flow rate (unit: mL/min), while the latter deals with absolute volume expansion and rate (unit: /min). The same Hb-derived plasma dilution is still used to indicate the volume expansion of *V*_*c*_ resulting from IV infusions (59). The micro-constant model is preferred over the clearance model as fluid volumes within the different body fluid spaces are now directly apparent and unequal fluid distribution across *V*_*c*_ and *V*_*p*_ compartments can be further investigated (30, 33).

With the evolution of VK models over time, many parameters have been represented differently, and so it is important to review the literature with an eye to this evolution to not be confused by the changes in symbols and parameters. This is further complicated by the use of proprietary symbols and expressions between PK computer software and PK studies (75). This has created a great degree of confusion and ambiguity, making PK and VK difficult to understand. Consistency in the use of symbols, units, and nomenclature would hopefully clarify the precise meaning of the term or concept as defined, curtail erroneous interpretation, and allow meaningful comparison between studies.

The following symbols and abbreviations are deduced to be equivalent dependent on whether a clearance model or a micro-constant model is presented: *V*_1_ = *V*_*c*_, *V*_2_ = *V*_*t*_ = *V*_*p*_, *v*_1_ = *v*_*c*_, *v*_2_ = *v*_*t*_ = *v*_*p*_, *k*_*i*_ = *R*_0_, *k*_*b*_ = *Cl*_0_ = *k*_0_, *k*_*r*_ = *Cl* ≅ *k*_10_, and *k*_*t*_ = *Cl*_*d*_ ≅ *k*_12_ and *k*_21_ (25, 32, 59, 67). To improve uniformity and clarity, the parameters, symbols, and abbreviations analogous to those used in PK (75, 76) should be used. Comparison of conventional PK and analogous VK parameters are summarized in Table 1.

**Table 1:** Comparison of conventional pharmacokinetic and analogous volume kinetic parameters.

**Parameters**	**Pharmacokinetics**	**Volume kinetics^a^**
Modeled entity (unit)	Mass, *X* (mg)	Volume expansion, (*v* − *V*) (mL)
Quantity in body (unit)	Amount, *A* = *C*_*p*_ × *V*_*d*_ (mg)	Volume expansion, $\left(v-V\right)=\frac{Hb/Hb_{\left(t\right)}-1}{1-\;HCT}\times V$ (mL)
Primary input variable (unit)	Concentration, $C_{p}=\frac{A}{V_{d}}$ (mg/mL)	Plasma dilution, $\frac{\left(v-V\right)}{V}=\frac{Hb/Hb_{\left(t\right)}-1}{1-\;HCT}$ (dimensionless)
Key parameters of interest (unit)	Volume of distribution, $V_{d}=\frac{A}{C_{p}}$ (mL) Total clearance, *CL*_*T*_ (mL/min)	Volume of body fluid space, *V* or *V*_*c*_ (mL) Clearance model: *Cl*, *Cl*_*d*_ (mL/min) Micro-constant model: *k*_10_, *k*_12_, *k*_21_ (/min)
Intravenous infusion rate (unit)	*R*_0_ (mg/min)	*R*_0_ (mL/min)
Zero-order elimination (unit)	Fixed amount of substance eliminated per unit time: e.g., Ethanol	Clearance model: Basal fluid losses, *Cl*_0_ (mL/min) Micro-constant model: Basal fluid losses, *k*_0_ (mL/min)
First-order elimination (unit)	Constant fraction of substance eliminated per unit time: e.g., Most drugs *k*_10_ (/min)	Clearance model: *Cl* (mL/min) Micro-constant model: *k*_10_ (/min)
Intercompartmental rate constants (unit)	*k*_12_, *k*_21_ (/min)	Clearance model: Distribution clearance, *Cl*_*d*_ (mL/min) Micro-constant model: *k*_12_, *k*_21_ (/min)
Change in quantity per time (unit)	$\frac{dA}{dt}=R_{0}-k_{10}\times C_{p\left(t\right)}$ (mg/min)	Clearance model: $\frac{dv}{dt}=R_{0}-Cl\times \frac{\left(v_{\left(t\right)}-V\right)}{V}$ (mL/min) Micro-constant model: $\frac{dv}{dt}=R_{0}-k_{10}\times \left(v_{\left(t\right)}-V\right)$ (mL/min)
Rate of elimination (unit)	*R*_*el*_ = *CL*_*T*_ × *C*_*p*(*t*)_ (mg/min)	Clearance model: $R_{el}=Cl\times \frac{\left(v_{\left(t\right)}-V\right)}{V}$ (mL/min) Micro-constant model: _*R*_*el*_ = *k*10_ × (*v*_(*t*)_ − *V*) (mL/min)
Renal clearance (unit)	$CL_{R}=\frac{\text{Amount in urine}}{AUC\text{ for plasma conc}.}$ (mL/min)	Clearance model, *Cl*≅*Cl*_*R*_: $Cl_{R}=\frac{\sum urine\;volume}{AUC\text{ for plasma dilution}}$ (mL/min) Micro-constant model: $Cl_{R}=\frac{\sum urine\;volume}{AUC\text{ for volume expansion}}$ (mL/min)
Elimination half-life (unit)	$t_{1/2}=\frac{0.693\times \;V_{d}}{CL_{T}}$ (min)	Clearance model: $t_{1/2}=\frac{0.693\times V}{Cl_{R}}$ (min)

a *Expressions for 1-VOFS kinetic model parameters are presented. Expressions for 2-VOFS kinetic model parameters are similar by substituting V and v with V_c_ and v_c_, respectively*.

*X, mass; A, amount; C_p_, plasma drug concentration; V_d_, volume of distribution; v, volume of expanded body fluid space; V, volume of expandable body fluid space; Hb, hemoglobin; Hb_(t)_, hemoglobin at any time t; HCT, hematocrit; V_c_, volume of expandable central body fluid space; CL_T_, total clearance; Cl, first-order clearance; k_10_, first-order elimination rate constant; Cl_d_, distribution clearance; k_12_, central to peripheral intercompartmental rate constant; k_21_, peripheral to central intercompartmental rate constant; R_0_, intravenous infusion rate; Cl_0_ or k_0_, zero-order basal fluid losses; R_el_, rate of elimination; v_(t)_, volume of expanded body fluid space at any time t; C_p(t)_, plasma drug concentration at any time t; ≅, approximately equal to; ∑, sum of; CL_R_ or Cl_R_, renal clearance; AUC, area under concentration-time curve; t_1/2_, half-life (31–33, 75, 76)*.

## Two-Volume Fluid Space Kinetic Model

Most IV fluids can be modeled using a 2-VOFS kinetic model (33, 55), also referred to as the bi-exponential model. The 2-VOFS kinetic model has been reported to best describe the effects of fluid therapy in human patients receiving crystalloid infusions during surgery, general anesthesia, dehydration, and hypovolemia (32), but does not always seem to describe the situation in healthy human volunteers (26, 27, 68) likely due to the rapid elimination of crystalloids in healthy awake states.

**Figure 2:**
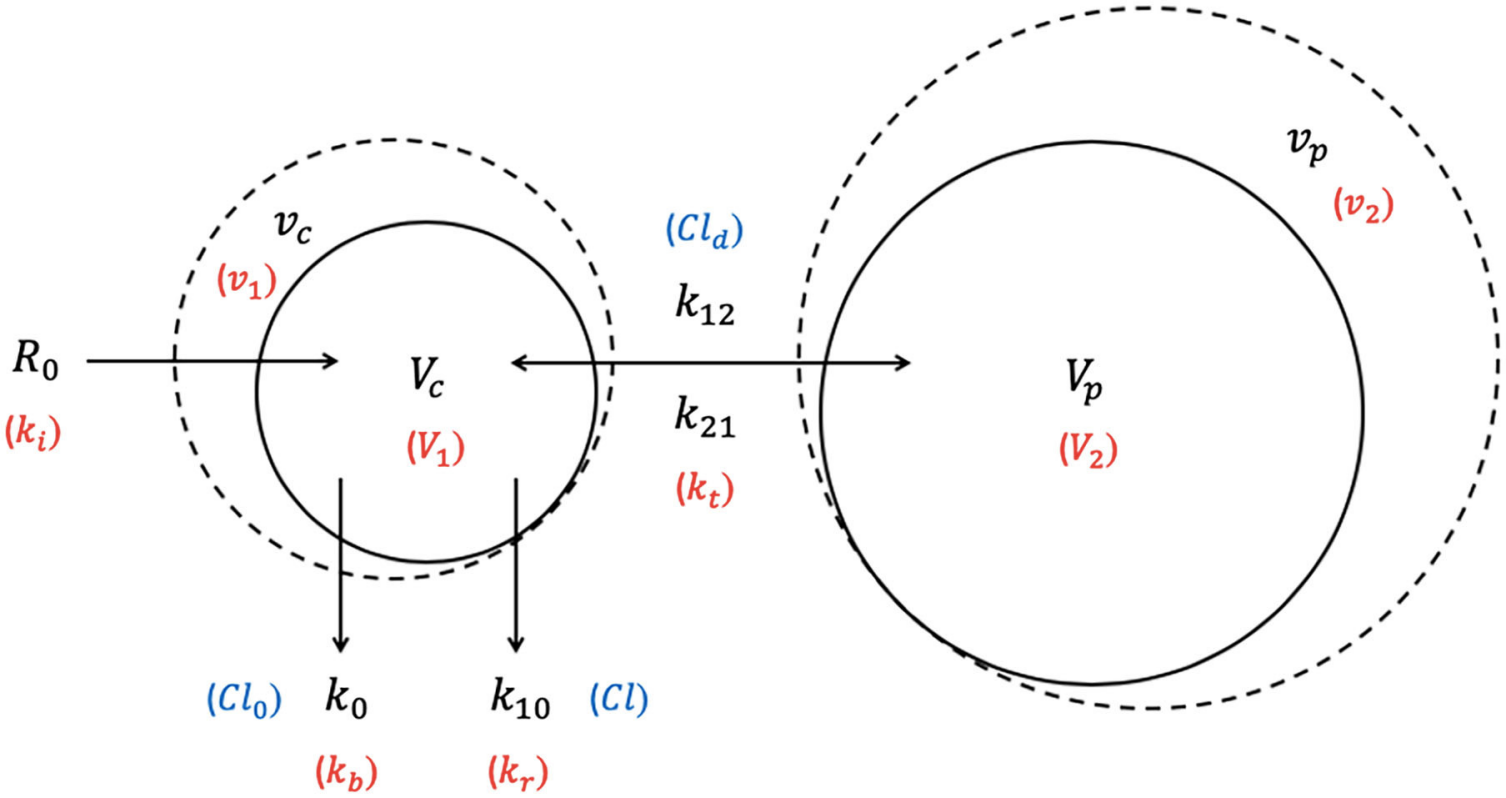
Two-volume fluid space (2-VOFS) kinetic model. Adapted from previous VK work (25, 26, 32, 67, 77–79). The symbols in black represent the current micro-constant model (67, 77, 78), whereas the symbols within red parentheses represent the original model (25, 26) and the symbols within blue parentheses represent the clearance model (32, 79).

The 2-VOFS model, as illustrated by Figure 2, suggests that IV fluid administered at a certain rate (*R*_0_) would expand the volume of a central body fluid space (*V*_*c*_) to a larger volume (*v*_*c*_) (32). As transcapillary hydrostatic and colloid osmotic pressure changes, the fractional plasma dilution ($\frac{v_{c}-V_{c}}{V_{c}}$) (26, 32) or absolute volume expansion (*v*_*c*_ − *V*_*c*_) (67, 77, 78) indicate distribution of fluid to a peripheral body fluid space (*V*_*p*_) which in turn expands to a greater volume (*v*_*p*_). These central and peripheral body fluid spaces attempt to retain their respective initial volumes to maintain homeostasis (26, 32). The net rate of fluid flux between the central and peripheral body fluid spaces is governed by the volume expansion difference between *V*_*c*_ and *V*_*p*_ multiplied by the distribution clearance (*Cl*_*d*_) in the clearance model (32, 79), or the intercompartmental rate constants (*k*_12_, *k*_21_) in the micro-constant model (67, 77, 78). In the micro-constant model, intercompartmental rate constants can be further categorized into central to peripheral intercompartmental rate constant (*k*_12_) and peripheral to central intercompartmental rate constant (*k*_21_) akin to PK analysis (67, 77).

Fluid elimination occurs from the central body fluid space via two mechanisms: (a) baseline water loss which is accounted for by zero-order elimination (*Cl*_0_ or *k*_*b*_) often pre-set between 0.3 and 0.5 mL/min in humans (66, 68, 80), and (b) dilution-dependent, primarily renal excretion governed by first-order elimination (*Cl* or *k*_10_) (32). The rate of elimination is given by the product of fractional plasma dilution ($\frac{v_{c}-V_{c}}{V_{c}})$ (26, 32) or absolute volume expansion (*v*_*c*_ − *V*_*c*_) (67, 77, 78), and the first-order elimination clearance (*Cl*) or elimination rate constant (*k*_10_) (32).

The unknown parameters in the 2-VOFS kinetic model that are estimated by non-linear least-squares regression are *V*_*c*_, *Cl*_*d*_ (or *k*_12_ and *k*_21_), and *Cl* (or *k*_10_) (31). If urine output is measured, *k*_0_ can even be estimated and includes all other body fluid not allocated by the VK model along with the basal fluid loss (32, 60). The first-order elimination (*Cl* or *k*_10_) is then set to a fixed value determined by the total urinary excretion obtained. In this scenario, *Cl* is analogous to the renal clearance which is calculated by dividing the urine output by the integral of the plasma dilution-time curve, also known as the area under concentration-time curve (*AUC*), following the assumption that half of the *Cl*_0_ or *k*_0_ due to insensible fluid losses appears as urine (60). The following differential equations describe the clearance model for the 2-VOFS kinetic model (30):

(1) $$\begin{array}{l} \frac{dv_{c}}{dt}=\;R_{0}-\;Cl_{0}-\;Cl\frac{\left(v_{c}-\;V_{c}\right)}{V_{c}}\\ \;-\;Cl_{d}\left[\frac{\left(v_{c}-\;V_{c}\right)}{V_{c}}-\;\frac{\left(v_{p}-\;V_{p}\right)}{V_{p}}\right] \end{array}$$

(2) $$\begin{array}{l} \frac{dv_{p}}{dt}=Cl_{d}\left[\frac{\left(v_{c}-\;V_{c}\right)}{V_{c}}-\;\frac{\left(v_{p}-\;V_{p}\right)}{V_{p}}\right] \end{array}$$

The differential equations describing the micro-constant model for the 2-VOFS kinetic model are as follows (30):

(3) $$\begin{array}{l} \frac{dv_{c}}{dt}=\;R_{0}-\;k_{0}-\;k_{10}\left(v_{c}-V_{c}\right)\\ \;-\;k_{12}\left(v_{c}-V_{c}\right)+k_{21}\left(v_{p}-V_{p}\right) \end{array}$$

(4) $$\begin{array}{l} \frac{dv_{p}}{dt}=\;k_{12}\left(v_{c}-V_{c}\right)-k_{21}\left(v_{p}-V_{p}\right) \end{array}$$

By comparing Equation (1) for the clearance model with Equation (3) for the micro-constant model, net *Cl*_*d*_ is obtained as the product of *V*_*c*_ × *k*_12_ and *V*_*p*_ × *k*_21_ (30). Similarly, *Cl* is the product of *V*_*c*_ × *k*_10_ (30).

## One-Volume Fluid Space Kinetic Model

Intravenous fluid plasma dilution-time profiles may not always display the bi-exponential form of a 2-VOFS kinetic model and sometimes statistically fit the 1-VOFS kinetic model better (32). A common scenario where this occurs is when crystalloids are eliminated rapidly from the body (27, 68), leading to an increased *Cl* : *Cl*_*d*_ (or *k*_10_ : *k*_12_ and *k*_21_) ratio. This reduces the time for full *V*_*p*_ expansion prior to elimination, therefore *V*_*c*_ and the partially expanded *V*_*p*_ fuse into a moderate size single body fluid space, allowing 1-VOFS kinetic model to sufficiently describe these dilution-time profiles (32). In addition, colloidal solutions with high molecular weight polymers that do not distribute extensively into the extravascular tissues should theoretically be described by the 1-VOFS kinetic model (31).

**Figure 3:**
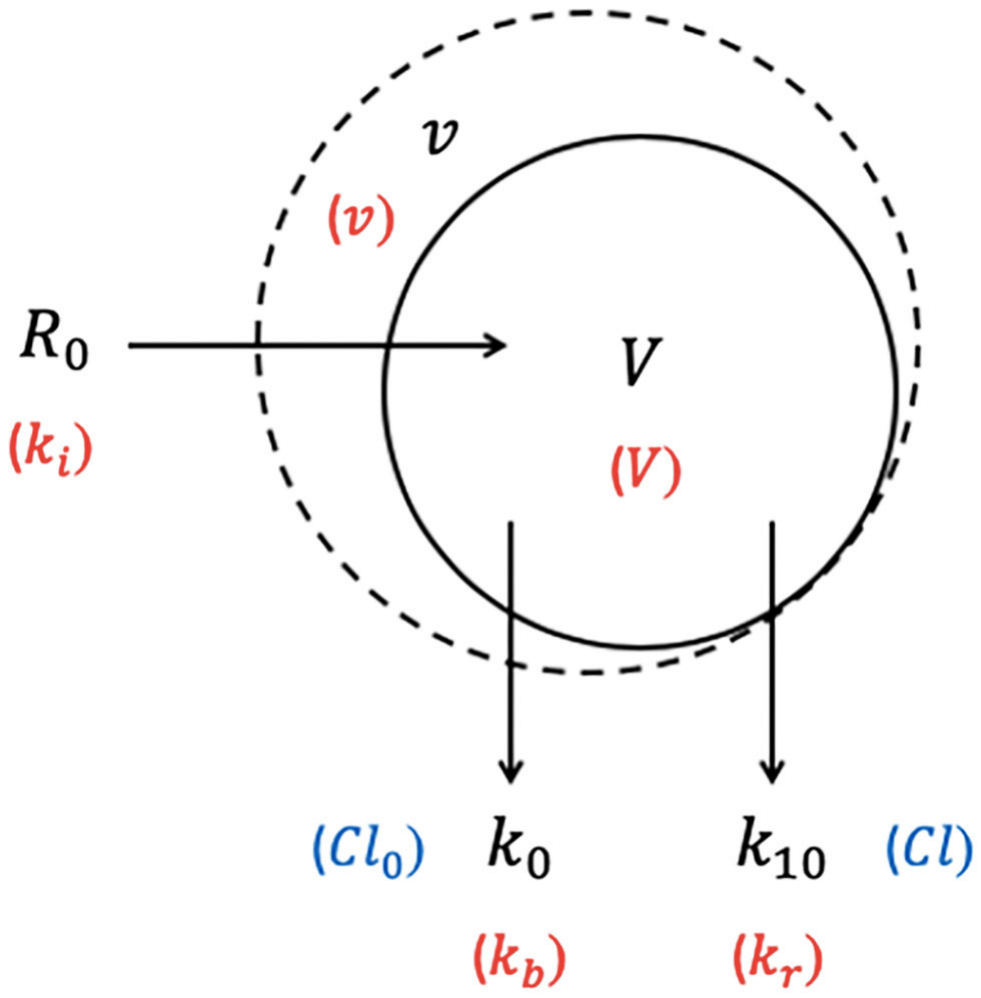
One-volume fluid space (1-VOFS) kinetic model. Adapted from previous VK work (25, 26, 32, 67, 77–79). The symbols in black represent the current micro-constant model (67, 77, 78), whereas the symbols within red parentheses represent the original model (25, 26) and the symbols within blue parentheses represent the clearance model (32, 79).

The 1-VOFS kinetic model, also known as the mono-exponential model, is illustrated by Figure 3. As the name suggests, IV fluid administered at a given rate (*R*_0_) is thought to expand the volume of a single body fluid space (*V*) to a larger volume (*v*) (32). Elimination of fluid still occurs via basal fluid losses (*Cl*_0_ or *k*_*b*_) and a dilution-dependent, primary renal excretion governed by a first-order elimination (*Cl* or *k*_10_) (32). The unknown parameters in the 1-VOFS kinetic model that are estimated by non-linear least-squares regression include *V* and *Cl* (or *k*_10_) (31). The fractional plasma dilution ($\frac{\left(v\;-\;V\right)}{V}$) in the clearance model or absolute volume expansion (*v* − *V*) in the micro-constant model, is obtained from plasma dilution computed from serial Hb concentration. Similar to the 2-VOFS kinetic model, *Cl*_0_ and *Cl* can be estimated through VK modeling or calculated if urine output is available. The following differential equation describes the clearance model for the 1-VOFS kinetic model (32, 79):

(5) $$\begin{array}{l} \frac{dv}{dt}=R_{0}-Cl_{0}-Cl\frac{\left(v\;-\;V\right)}{V} \end{array}$$

In comparison, the differential equation that describes the micro-constant model for the 1-VOFS kinetic model is as below (67, 77, 78):

(6) $$\begin{array}{l} \frac{dv}{dt}=R_{0}-k_{0}-k_{10}\left(v-V\right) \end{array}$$

## Mass Balance Principles

The volumes of individual body fluid compartments are conventionally estimated using external indicator-dilution techniques. Some examples include but are not limited to, deuterium oxide (81) or radioactive tritium oxide (82) for total body water determination; bromide (83, 84) for extracellular fluid volume determination; and radio-iodinated serum albumin (18), Evans Blue dye (18, 36), or indocyanine green dye (17, 18, 38) for plasma volume determination. Based on the law of mass conservation, indicator-dilution techniques fulfill the mass balance principle at steady-state, whereby the total indicator mass following distribution in the fluid compartment will remain the same as the total mass injected into the compartment (80, 85). Hence, the volume of a body fluid compartment can be estimated provided that the indicator's concentration following homogenous mixing can be accurately determined (84).

Although exogenous indicator-dilution techniques are considered the conventional “gold standard” method for volume determination (17, 86), they can result in hypersensitivity reactions and potential errors as a result of indicator extravasation, plasma turbidity, Hb contamination, or controversial back-extrapolation methods (18, 87–90). Radioactive isotopes are cumbersome to use and there is a significant time delay for exogenous indicators to equilibrate to a steady-state distribution (80, 84). This precludes the ability to distinguish between the distribution and elimination phases (34, 67) and does not permit analysis of acute dynamic fluid shifts (36).

In 1987, an endogenous indicator-dilution technique using blood Hb concentration was introduced as an accurate method for estimation of BV changes following validation with the radio-iodinated serum albumin technique (21). Endogenous Hb is confined solely within the plasma space, not associated with any time-delay for steady-state equilibrium, much easier to use, and widely measurable in routine clinical laboratories (21, 67). Therefore, serial measurement of Hb concentrations following IV fluid administration can be used to quantify plasma dilution in the blood and represent acute dynamic fluid shifts within the body (21). The mass balance technique using serial Hb dilution requires the assumption that total body Hb remains constant and homogenously distributed within the circulation at all times, except during sampling losses or hemorrhage to which correction for Hb losses can be made (21, 38).

## Hemoglobin-Derived Plasma Dilution

Plasma and Hb are the main components of the central body fluid space, and the change in *V* or *V*_*c*_ is directly proportional to plasma dilution (26). Therefore, serial measurement of Hb concentrations following IV fluid administration can be used to quantify plasma dilution in the blood. Hemoglobin dilution must be converted to the corresponding plasma dilution considering that extracellular fluid, and not red blood cells (RBC), are expanded by the infused fluid. Moreover, PV is the actual entity that distributes across body fluid spaces and from which water is ultimately eliminated from the body. Plasma dilution, which equals the dilution of *V* or *V*_*c*_, is used to quantitate fluid volume load. As such, the concentration of IV fluid, or volume expansion, is expressed in terms of Hb-derived plasma dilution as a function of time (32), given by the reference Equation (7). This reference equation can be applied directly into the non-linear least square regression curve-fitting procedure if blood sampling and hemorrhage are negligible.

(7) $$\begin{array}{l} Plasma\;dilution,\;\frac{v_{\left(t\right)}-V}{V}=\frac{\frac{Hb_{\left(0\right)}-Hb_{\left(t\right)}}{Hb_{\left(t\right)}}}{1-HCT}=\frac{\frac{Hb_{\left(0\right)}}{Hb_{\left(t\right)}}-1}{1-HCT} \end{array}$$

Simultaneously measured RBCs are diluted similarly as Hb during IV fluid loading but quantified using a different technique by the automated hematology analyzer. Therefore, if available, RBC count and RBC mass could also be included in the curve-fitting procedure to improve precision and accuracy (32), although the author's (RGH) experience with this adaptation yielded negligible effect. The same calculations using the reference Equation (7) are performed for RBC count and RBC mass over time, and the mean value for Hb-derived plasma dilution and RBC-derived plasma dilution can be used as the overall plasma dilution (91). Calculated hematocrit value obtained from automated hematology analyzers is preferred over packed cell volume for plasma dilution determination as the latter frequently overestimates true RBC fraction due to incomplete microhematocrit centrifugation compaction (80).

## Corrections for Hemoglobin Loss

Serial blood sampling can result in sufficient loss of Hb creating a “false” dilution that is unrelated to IV fluid therapy. Therefore, blood loss and sampled volume should be corrected prior to the curve-fitting procedure. The calculations to correct for Hb loss and alteration in erythrocyte size consider the PV expansion instead of plasma dilution. To account for sampling loss, change in BV is calculated by estimating the total Hb mass in the circulation from which blood loss is subtracted (21). An assumption of initial BV has to be preset to time 0 (31). The total baseline Hb mass (*MHb*_(0)_) is first obtained, then the total Hb mass corrected for sampling at any time (*t*), followed by the expanded BV corrected for sampling at any time (*t*) (32):

(8) $$\begin{array}{l} MHb_{\left(0\right)}=BV_{\left(0\right)}\times Hb_{\left(0\right)} \end{array}$$

(9) $$\begin{array}{l} MHb_{\left(t\right)corr.}=MHb_{\left(0\right)}-\left[\left(sampled+bled\right)\;volume\times Hb_{\left(t\right)}\right] \end{array}$$

(10) $$\begin{array}{l} BV_{\left(t\right)corr.}=\frac{MHb_{\left(t\right)corr.}}{Hb_{\left(t\right)}} \end{array}$$

Changes in BV at any time (*t*) can be provided by Equation (11), and the BV expression can be easily transformed to PV using Equation (12):

(11) $$\begin{array}{l} \Delta BV=BV_{\left(t\right)corr.}-BV_{\left(0\right)} \end{array}$$

(12) $$\begin{array}{l} PV=BV\times \left(1-HCT\right) \end{array}$$

(13) $$\begin{array}{l} PV_{\left(t\right)}=BV_{\left(t\right)}\times \left[1-HCT\times \frac{Hb_{\left(t\right)}}{Hb_{\left(0\right)}}\right] \end{array}$$

Corrected plasma dilution,

(14) $$\begin{array}{l} \frac{v_{\left(t\right)}-V}{V}=\frac{PV_{\left(t\right)}-PV}{PV} \end{array}$$

The relationship between baseline Hb (*Hb*_(0)_) and diluted Hb at any time (*t*) is written as $\frac{Hb_{\left(0\right)}}{Hb_{\left(t\right)}}$ in the reference Equation (7), whereas the inverse relationship is used in Equations (13, 14) (32). The lower Hb concentration must be placed as the denominator of the ratio in the reference Equation (7) to arrive at a correct proportion between changes in Hb and water volume.

The degree of error introduced by the wide range of initial BV has been shown to be forgiving (66), however the error related to frequent blood sampling and the use of successive Hb ratio for volume estimation should not be ignored as the volume-estimation errors can increase exponentially (92). Given the importance of an accurate baseline value, averaging duplicate and triplicate Hb measurements on the initial blood sample is strongly recommended, and the percent coefficient of variation for the entire sampling and analysis should be determined (92). Duplicate Hb measurements for subsequent blood samples throughout the studies have been attempted by the author (RGH) with marginal improvement to VK analysis.

## Corrections for Alternation in Mean Corpuscular Volume and Osmotic Fluid Shift

When investigating IV fluids that alter plasma osmolality, for example hypertonic saline (HS) or glucose infusions, a correction for changes in mean corpuscular volume should also be considered. Mean corpuscular volume can be altered in humans following infusion of HS because of osmotically induced decreases in intracellular water, this has also been reported in dogs and cats (93). Changes in mean corpuscular volume can be considered by the addition of a term for the relationship between the baseline (*MCV*_(0)_) and at any time (*t*) into Equation (13) above:

(15) $$\begin{array}{l} PV_{\left(t\right)}=BV_{\left(t\right)}\times \left[1-HCT\times \frac{Hb_{\left(t\right)}}{Hb_{\left(0\right)}}\times \frac{MCV_{\left(t\right)}}{MCV_{\left(0\right)}}\right] \end{array}$$

If HS or glucose solutions are infused, an osmotic fluid shift theoretically occurs across the cell membrane and water moves from the intracellular fluid space (~40% of BW) to the extracellular fluid space (~20% of BW) (80). Using species-specific baseline serum osmolality (*Osm*_(0)_), which is ~295 mOsm/kg for humans (32), ~317 mOsm/kg for cats (94), and ~302 mOsm/kg for dogs (95), the translocated volume (*f*_*t*_) can be obtained from Equation (16) (37, 68):

(16) $$\begin{array}{l} \frac{BW\times 20\%\times Osm_{\left(0\right)}+\text{infused osmoles}}{BW\times 20\%+f_{t}+\text{infused volume}}\\ \;=\frac{BW\times 40\%\times Osm_{\left(0\right)}}{BW\times 40\%-f_{t}} \end{array}$$

The osmotic force diminishes progressively with subsequent amount of infused fluid; therefore, *f*_*t*_ should be entered as a linear function in the analysis process such that *f*_*t*_ at each point in time is governed by the total amount of infused fluid (32).

## Volume Kinetic Models in Physiological Contexts

Recall that PK compartment models and VK kinetic models are mathematical concepts that describe body spaces which a substance or fluid appears to occupy, but may not correspond to an actual anatomical space or physiological volume (31, 54). In PK analysis, *V*_*d*_ is theoretical and does not equate to a real volume (53, 54). A substance could have a large *V*_*d*_ that substantially exceeds the total body volume (54), implying that it is highly distributed in tissues (53). The smallest *V*_*d*_ is the PV (51) which suggests that the substance is poorly distributed and confined to the plasma (53). Similarly, this concept applies to *V* and *V*_*c*_ which substitute *V*_*d*_ in VK analysis.

In healthy human VK studies, the size of *V*_*c*_ for crystalloids using a 2-VOFS kinetic model has been reported as 3–4 L (4–6% of BW) (26, 31, 60, 68), which is similar to the expected (80) and measured human PV (69). However, when a 1-VOFS kinetic model is used, the size of *V* is usually twice the size of PV (59, 60, 68) as it combines both the plasma volume and approximately half of the interstitial fluid volume. As is expected for colloids, the size of *V* of a 1-VOFS kinetic model was found to be analogous to the PV (59).

The size of *V*_*p*_ in healthy adult humans was discovered to be 6–8 L (8–11% of BW) (26, 68, 96), which is smaller than the expected size of the interstitial fluid space (11 L or 15% of BW) (80). The *V*_*p*_ encompasses about 66.6% of the interstitial fluid space since bones, cartilage, dense connective tissues, and organs with a tight fibrous capsule are either not expandable or minimally expandable by fluid (26, 34, 96). Unlike the conventional exogenous indicator-dilution techniques, VK only highlights expandable body fluid spaces (26). Some interstitial tissues have increased compliance for volume expansion while others require very elevated fluid pressure before expansion can occur (97, 98). The *V*_*p*_ was discovered to be larger when massive fluid infusions beyond what is routinely safe in clinical practice were administered to sheep (39). The precision of *V*_*p*_ is often lower than *V*_*c*_ (26, 32, 60) but is less crucial since the size of *V*_*p*_ does not contribute significantly to interpretable knowledge (67). So far, estimates of *V*_*p*_ that are significantly smaller than *V*_*c*_ have not been reported (67).

In healthy humans, there should be no restriction on fluid movement into the peripheral body fluid space or return to the central body fluid space. Distribution clearance (*Cl*_*d*_) or the intercompartmental rate constants (*k*_12_, *k*_21_) can be appointed the same value for bi-directional flow based on the assumption that fluid is equally prone to flow in either direction (32). Alternatively, uneven distribution can be analyzed using the micro-constant model by splitting the intercompartmental rate constants into *k*_12_ and *k*_21_, which could serve to quantify the accumulation of peripheral edema (30, 36, 38). Fluid exchange is considered normal with *k*_12_ that is twice as high as *k*_21_ (33, 71, 77).

According to the model, the parameter *Cl* (or *k*_10_) that is estimated by non-linear least square regression should approximate the renal clearance (*Cl*_*R*_), since the ultimate destiny of IV fluid is elimination via the kidneys (31). It is, however, important to remember that parameter estimates generated from VK modeling are not absolute measurements of physiologic body fluid variables, but rather represent estimates of abstract concepts which provide insights into the body's handling of IV fluid (31). The first-order elimination rate constant has been found to correlate well with urine output in human volunteers receiving isotonic crystalloids (26, 27, 60, 66) and normovolemic conscious sheep (36). However, a discrepancy in measured and estimated values resulting in underestimation of *V*_*p*_ was reported in sheep during general anesthesia receiving positive pressure ventilation (36). The most common way to calculate the elimination half-life (*t*_1/2_) of infused fluid is by utilizing *Cl*_*R*_ which is obtained by dividing urine output with the AUC for the fractional plasma dilution or absolute volume expansion in the clearance or micro-constant model, respectively (30).

## Clinical and Research Applications

The use of VK overcomes the limitations of external indicator-dilution techniques for physiologic spaces estimation or measurements of hemodynamic end-points (37). An external indicator distributes within conventional physiological body fluid spaces but may not reflect the effects of IV fluid under dynamic, non-steady state circumstances. Hemodynamic end-points undeniably provide a different set of useful information, but fundamental information about fluid shifts, functional body fluid space volumes, or mechanisms behind differences in fluid dynamics are not assessable. Although VK fluid space models and parameter estimates are less intuitive and describe fluid handling without precisely measuring physiologic spaces, interpreting them in light of mass balance analysis may improve understanding and describe changes in traditional physiologic compartments that are more familiar to both scientist and clinicians (38). The clinical implications of VK analysis in healthy and sick animals are summarized in Table 2.

**Table 2:** Clinical implications of volume kinetics in healthy and sick animals.

	**Clinical implications**
Healthy animals	1. The distribution and elimination of isotonic crystalloid fluid bolus in sheep are markedly different under awake and anesthesized states (36, 38). a. Isotonic crystalloid solution is rapidly eliminated from the intravascular space via urinary excretion in awake sheep (36, 38). b. Isoflurane anesthesia significantly decreases urinary excretion (fluid elimination), leading to peripheral fluid distribution and accumulation (36, 38).
	2. Large, rapid isotonic crystalloid fluid boluses exceeding renal excretory capacity contribute to peripheral fluid accumulation in sheep (39).
	3. Vasoactive agents alter the distribution and elimination of isotonic crystalloid fluid in healthy awake sheep (43, 45). a. α_1_-adrenergic stimulation reduces PV expansion by increasing fluid elimination (urinary excretion) and peripheral fluid distribution (43, 45). b. β_1_-adrenergic stimulation reduces fluid elimination and peripheral fluid distribution, thereby improving hemodynamics through more effective PV expansion (43, 45).
	4. Mannitol infusion has lower PV expansion effects and contributes to peripheral fluid accumulation in pigs due to higher fluid elimination (osmotic diuresis) and fluid distribution down the osmotic gradient (natriuresis-induced and dilutional hyponatremia) (35).
Sick animals	1. Early normotensive *Escherichia coli* endotoxemia changes the distribution and elimination of balanced crystalloid fluid bolus in rabbits (34).
	2. Early or late sepsis induced by *Pseudomonas aeruginosa* bacteremia did not change the PV expansion, distribution, and elimination of a single isotonic crystalloid fluid bolus in sheep (40).
	3. Vasoactive agents alter the distribution and elimination of isotonic crystalloid fluid in septic anesthetized sheep (43, 45), similarly to healthy awake sheep (43, 45). a. α_1_-adrenergic stimulation worsens peripheral fluid accumulation in septic anesthetized sheep, as urinary excretion is not increased to the same extent as in healthy awake sheep (43, 45).

### Published Work in Human Literature

There are now more than 60 research publications in human medicine describing the VK of isotonic crystalloids, balanced crystalloids, hypertonic solutions, and colloids under diverse surgical and physiological conditions (30–33). Although first introduced two decades ago, VK studies continue to appear in the scientific literature (70–74, 99–102) and the research method is starting to gain recognition (15, 16), especially in light of the increasing debate on the safety and changing practices of fluid therapy.

The first *in vivo* VK study described the effects of commonly used equipotent IV fluid boluses on the expandable fluid spaces in healthy male human volunteers (mean 80 kg) (26). Balanced crystalloid (25 mL/kg acetated Ringer's solution over 30 min) was found to generate the largest plasma dilution, while the dilutions corresponding to colloidal solution (5 mL/kg 6% Dextran 70 in 0.9% NaCl over 30 min) and HS (3 mL/kg 7.5% NaCl over 30 min) were comparable but with a longer duration of effect (26). Urinary excretion was not measured, and *k*_10_ (reported as *k*_*r*_ in the original model) was computed by VK modeling (26). This work was further extended to investigate the effects of different balanced crystalloid infusion rates (25 mL/kg acetated Ringer's solution over 15, 30, 45, and 80 min) and volumes (12.5 mL/kg acetated Ringer's solution over 30 min) in healthy female human volunteers (mean 60 kg) (27). Acetated Ringer's solution was discovered to have a more effective and extended plasma expansion effect when infused over a longer time period, and reached a maximum effect of 36% volume expansion (550 mL in an adult human) regardless of infusion rates (27).

Over the years, VK analysis has provided some mechanistic explanations as to why and when patients are sensitive to large volumes of fluid (103). Slow fluid distribution to the peripheral compartment has resulted in a 50–75% larger plasma dilution during a crystalloid infusion than would be expected if the distribution had been immediate (32). In healthy conscious volunteers, isotonic balanced crystalloids are reported to take up to 25–30 min to distribute following rapid IV infusion (25 mL/kg over 30 min) (32). Due to this lag time for crystalloids to equilibrate between plasma and interstitium (66), PV expansion during the actual infusion is much larger than the commonly suggested expansion of 20–25% of the infused volume (32). Several studies have shown that the immediate PV expansion can be substantial. In a group of normovolemic human volunteers, 50% of a 2 L acetated Ringer's solution given over 20 min was retained within the intravascular compartment at the end of the infusion (66). In a study where acetated Ringer's solution was infused continuously throughout transurethral resection of the prostate in male adults under general anesthesia (104), fluid retention averaged 60% of the infused volume. The fraction of infused fluid that persists in the intravascular compartment is higher for slower infusions (27). Therefore, crystalloids may be better PV expanders than currently acknowledged, provided that the infusion is continuous and not administered as a bolus.

Volume kinetic analysis has also been used to describe the distribution and elimination of isotonic and hypertonic fluids within the body (68). When IV administration of 25 mL/kg of 0.9% NaCl was used as a reference fluid and compared to an equal volume of lactated Ringer's solution, acetated Ringer's solution, 5 mL/kg of 7.5% NaCl, and 3 mL/kg of 7.5% NaCl in 6% dextran solution (HSD), all infused over 30 min, plasma dilution efficiency according to the AUC of dilution-time profiles was found to be 0.88, 0.91, 3.97, and 7.22, respectively in 10 healthy male volunteers (mean 81 kg) (68). Based on VK analysis and simulation, the strength of these respective fluids to dilute the plasma by 20% within 30 min was 0.94, 0.97, 4.44, and 6.15 times greater than of 0.9% NaCl, respectively (68). Urinary excretion was found to be 1.8 times and 2.7 times larger than the infused volume of HS and HSD, which the authors attributed to natriuresis induced by sodium load (68). In this study, comparison between fluids was complicated by the need for several models, including a three-volume fluid space kinetic model for hypertonic fluid, to account for possible osmotic fluid shift (*f*_*t*_).

In a more recent study (59), the volume effects of 10 mL/kg of 6% HES 130/0.4/9:1 (Voluven®), 20 mL/kg of acetated Ringer's solution, and a combination of HES and acetated Ringer's solution administered 75 min apart was explored using VK in 10 healthy male volunteers (mean 79 kg). The kinetic models were successfully fitted to all experiments using the 1-VOFS kinetic model for HES and the 2-VOFS kinetic model for acetated Ringer's solution. The *V*_*c*_ for HES in both series of experiments was 3.14 L (~5% of BW), close to the PV estimated by anthropometry, with a *t*_1/2_ of 2 h (59) similar to the *t*_1/2_ reported in the product monograph (105). Hydroxyethyl starch was found to induce diuresis with 85% of the infused volume excreted as urine when administered alone (59). The *V*_*c*_ for acetated Ringer's solution averaged 4.88 L (~6.2% of BW) with a *t*_1/2_ of 88 min (59), similar to the *t*_1/2_ of 82 min reported with 2% dehydration induced by furosemide (79). The authors thus attributed the prolonged *t*_1/2_ to mild dehydration following an overnight fast. The *t*_1/2_of acetated Ringer's solution was otherwise reported to average 21 min in euhydrated conscious volunteers (79). When combined with HES, the distribution and elimination of acetated Ringer's solution occurred more slowly than in the single-infusion experiments (59).

Volume kinetic modeling has been used to evaluate IV fluid dynamics using different types of IV fluid solutions (26, 59, 68, 74) and rates (71) in various age groups (106, 107), as well as fluid shifts during inhalation anesthesia (99, 108), epidural anesthesia (109, 110), subarachnoid block (106, 108, 111–113), dehydration (78, 79, 114), hemorrhage (66, 73, 114), acute systemic inflammatory states (72), adrenergic influence (77), perioperative period (115), surgical trauma (96, 116), glucose supplementation (117–119), and pre-eclampsia (120). Through the various VK studies accumulated over the years, the *t*_1/2_ of various IV solutions can now be summarized and were discovered to be significantly variable governed by multiple factors such as the type of fluid and the patient's physiological conditions, further supporting the importance of clinical context (30). Of note, this body of work has been conducted exclusively by the same group of researchers, perhaps due to the intricacy of these analyses, and VK has not found widespread utilization by other researchers. Several international research teams have published on the development of locally modified mathematical fluid kinetic models (121–124) and external validation attempts of these models (125–127). Microcirculatory exchange models that predict fluid, protein, and small ion distribution in the vascular, interstitial, lymphatic, and intracellular compartments using mass balance equations for fluid and individual solutes along with auxiliary transport equations, instead of plasma dilution, have also been explored (22, 128). These model-predicted fluid volume changes were comparable to published experimental data (129) and clinical data obtained using segmental bioelectrical impedance analysis (22).

### Previous Work in Animal Research Models

To date, VK analysis has only been utilized in rabbits (34), pigs (35), and sheep (36–45) which served as experimental research models for human medicine. Sheep and pigs are common biomedical research models for the study of major human physiological systems including the cardiovascular, respiratory, and renal systems due to their well-defined anatomy, physiology, and large body size which permits frequent blood sampling as well as instrumentation with monitoring and sampling devices (130, 131). Translation of experimental work in animal models to humans is feasible, although some species differences exist, such as the reservoir function of the spleen. Thus far, VK modeling and analysis in sheep, rabbits, and pigs appears robust with clinically relevant results that are physiologically sound, providing credibility for wider application to other mammalian species that share similar cardiovascular-renal anatomy and physiologic characteristics.

The distribution and elimination of IV fluids have been investigated under the influence of inhalant anesthesia (36, 38, 42), various fluid infusion rates and duration (37, 39), hemorrhage (41), hypoproteinemia (44), sepsis (34, 40), and use of vasoactive agents (43, 45) in sheep, rabbit, and pig models. Aside from the aforementioned studies performed by the same group of researchers, there is only one veterinary study that investigated real-time IV fluid dynamics using a different method in healthy anesthetized dogs (20). Studies exploring the use of VK in companion animal species could not be identified following a search of the English literature from the MEDLINE® database using search terms such as “volume kinetic,” “fluid kinetic,” “fluid dynamics,” “fluid behavior,” “fluid distribution,” “fluid elimination,” “fluid half-life,” “canine,” “feline,” “dog,” “cat,” and “veterinary” via the PubMed®, Europe PubMed Central® (PMC), and Ovid® search engines. Therefore, VK studies in animal research models and the sole canine study form the only basis for additional discussion of fluid dynamics in veterinary medicine.

In 2002, Brauer et al. (36) investigated fluid dynamics of isotonic crystalloid infusion (25 mL/kg 0.9% NaCl over 20 min) in 6 normovolemic splenectomised sheep (mean 42 kg) while under isoflurane anesthesia and while conscious. Using a cross-over experimental design, the 2-VOFS kinetic model revealed that isotonic crystalloid solution was rapidly eliminated from *V*_*c*_ via urinary excretion (median 863 mL, range 604–1122 mL) in conscious sheep; however when anesthetized and mechanically ventilated, urinary excretion was markedly reduced (median 9 mL, range 4–150 mL) resulting in peripheral fluid accumulation (36). Unlike indicator-dilution techniques, VK analysis was able to demonstrate how fluid elimination from *V*_*c*_ transpired more rapidly than accounted for by urinary excretion, thus rather than being excreted, the fluid distributed and accumulated within the peripheral space (38). Plasma volume expansion was otherwise similar for both groups and reached 40–50% fluid efficacy at the immediate end of the infusion despite the marked differences in fluid distribution and elimination (36). Estimated VK parameters correlated well with parameters measured using the indicator-dilution technique with Evans blue dye in the conscious sheep and *V*_*c*_ was 1.6 L (~4% of BW) (36). However, clearance (reported as *k*_*r*_) predicted by VK analysis (58.5 mL/min) was significantly different from the manual calculation using median urinary excretion (0.6 mL/min) when sheep were anesthetized and mechanically ventilated (36). Estimates of *k*_*r*_ by VK analysis erroneously underestimated peripheral fluid accumulation by corresponding to the sum of urinary output and extravascular fluid accumulation (36). This led to the discovery that *k*_*r*_ simply reflects net fluid outflow from *V*_*c*_ and does not approximate urinary excretion of infused fluid as previously presumed (26, 27, 60, 109). Unfortunately, the combination of mechanical ventilation and isoflurane anesthesia in the study group precluded separation of individual intervention effects.

A subsequent experimental cross-over study by Connolly et al. (38) in 7 normovolemic splenectomised sheep (mean 28 kg) was designed to discern the effects of isoflurane anesthesia from mechanical ventilation. The study effectively demonstrated that isoflurane anesthesia alone was responsible for significantly decreased urinary excretion and promoting ECF accumulation during isotonic crystalloid volume loading (38). This study was conducted very thoroughly using 4 protocols: conscious spontaneous ventilation, conscious mechanical ventilation through tracheostomy tube, anesthetized spontaneous ventilation, and anesthetized mechanical ventilation. In this study, baseline PV (~5% BW) increased rapidly during the infusion of 25 mL/kg 0.9% NaCl over 20 min, achieving a 40% fluid efficacy at the immediate end of the infusion (38). This degree of immediate fluid expansion corroborated previous study findings in normovolemic sheep (36) and human volunteers (66). However, despite the initial PV expansion, rapid decline of volume expansion immediately ensued for a duration of 30 min (14% fluid efficacy) followed by a slower phase of decline until the end of the 3 h experiment (4% fluid efficacy) (38). Interestingly, the major findings of Connolly's study were contradictory to a common expectation that positive pressure ventilation alters circulating blood volume and peripheral fluid accumulation by impeding venous return and changing cardiac output (132). The absence of positive end-expiration pressure along with the use of normal tidal volumes (10–15 mL/kg) in euvolemic sheep with healthy compliant lungs may have caused inadequate dynamic changes in intrathoracic pressure and lung volume to induce appreciable cardiovascular effects and secondary fluid shifts. The physiologic mechanism by which isoflurane inhibits diuretic response to volume load and increases extravascular fluid retention has not been determined but reduced glomerular filtration rate and renal blood flow, as well as the involvement of antidiuretic hormone and atrial natriuretic peptide have been speculated to be possible contributing factors (38). A recent matched case-control study of 23 dogs, anesthetized for an elective orthopedic procedure receiving 10 mL/kg/hr of intravenous lactated Ringer's solution for 4 h, reported similar findings of decreased urine output production (<0.5 mL/kg/hr) and substantial peripheral fluid retention as evidenced by significant BW gain, positive fluid balance, as well as increased TBW and ECF volume measured using bioimpedance spectroscopy (133).

Brauer et al. (37) also studied the impact of infusion duration of 0.9% NaCl (6 mL/kg over 5 min, 24 mL/kg over 20 min) and 7.5% NaCl in 6% dextran solution (HSD) (4 mL/kg over 2 min and 20 min) in 6 conscious splenectomised sheep (mean 36 kg). The maximum arterial plasma dilution at the end of the 5 and 20 min 0.9% NaCl infusion were 10 and 22%, respectively. Meanwhile, maximum arterial plasma dilution after 2 and 20 min of HSD infusions were 24 and 21%, respectively. Therefore, the authors concluded that VK variables obtained during a short infusion can be used to predict the outcome of longer infusions, even if the longer infusion also delivers a larger volume (37). This work involving clinically relevant IV fluid doses suggests that VK modeling conforms to linearity such that kinetic parameters obtained can be used to simulate the outcome of other experiments. Subsequently, Svensen et al. (39) published their findings on the VK effects of various isotonic crystalloid infusion volumes and rates (25, 50, and 100 mL/kg of 0.9% NaCl over 20 min) in 6 conscious, splenectomised sheep. Elimination of isotonic crystalloid solution from *V*_*c*_ was found to be proportional to the magnitude of plasma dilution regardless of infused volumes and rates, and elimination occurs via expansion of *V*_*p*_ when renal excretion fails to increase in proportion to the volume of infused fluid (39). Therefore, large and rapid fluid boluses that exceed short-term renal excretory capacity contribute to peripheral fluid accumulation. This study also revealed that markedly supraphysiologic infusion doses exceed the limits of linearity of a VK model.

In 2010, Brauer et al. (44) found that severe acute hypoproteinemia induced by plasmapheresis does not reduce the PV expansion of isotonic crystalloid infusion (27 mL/kg 0.9% NaCl over 20 min) in non-hemorrhaged, non-splenectomised sheep. The depletion of mean total protein concentration from 5.4 to 2.5 g/dL following plasmapheresis resulted in a parallel reduction in mean plasma COP from 20 to 9.6 mmHg (44). Plasma volume expansion reached ~20% at the end of the infusion and stayed at 10–15% during the experiment (44). No difference in the PV expansion and cumulative urinary output was found between hypoproteinemic and normal sheep. This is contrary to the conventional physiological reasoning which predicts that a reduction in plasma oncotic pressure will increase fluid filtration, resulting in accumulation of interstitial fluid, reduced plasma protein concentration in capillary filtrate, and increased lymph flow. Splenic contraction and release of RBCs into the circulation affecting the accuracy of plasma dilution calculations were speculated to contribute to the findings however no evidence of hemoconcentration was documented (44), making this explanation less likely. It is intriguing to know if a plasma COP of 50% is sufficient to maintain normal fluid filtration.

The effects of systemic illness have also been explored using VK modeling. Svensen et al. (34) investigated the volume effect of a balanced crystalloid solution (25 mL/kg acetated Ringer's solution over 30 min) in 10 conscious rabbits (mean 4.4 kg) and found that early endotoxemia alters the body's handling of crystalloid solution (34). The expandable volume, *V*, which represents distribution to the ECF space decreased from 473 ± 37 mL (10% of BW) to 327 ± 54 mL (7.5% of BW), and the rate of elimination (reported as *k*_*r*_ in the original model) increased from 2.9 ± 0.5 to 5.9 ± 2.8 mL/min during early normotensive endotoxemia (34). Visual comparison of individual plasma dilution-time curves demonstrated markedly variable volume effect of acetated Ringer's solution following endotoxin administration. This study provided a glimpse into the net effects of various pathophysiological responses to endotoxemia on the volume effect of IV crystalloid solution, however individual responses that could account for the observed changes could not be isolated. The *V* of 10% BW obtained in healthy rabbits was in agreement with the size of *V* obtained in healthy humans (26, 27) but smaller than the anatomical ECF volume (~20% of BW) to which crystalloids were expected to distribute across.

Almost a decade later, Svensen et al. (40) found contradictory results in a population of septic sheep. The authors found that the distribution and elimination of isotonic crystalloid infusion (25 mL/kg 0.9% NaCl over 20 min) in 6 splenectomised sheep (mean 42 kg) were unchanged by early or late sepsis induced by *Pseudomonas aeruginosa* bacteremia (40). Plasma volume expansion was 312 ± 50 mL (~29.7% fluid efficacy), 386 ± 34 mL (~36.7% fluid efficacy), and 400 ± 51 mL (~38% fluid efficacy) in the control, early sepsis, and late sepsis group (40). Similar peak PV expansion, plasma dilution-time curves, and volume kinetic parameters were obtained for both the control and septic group. These results were contrary to clinical impressions that crystalloid fluid requirements are greatly increased in septic patients due to increased vascular permeability and rapid loss from the intravascular space. The elimination pattern of isotonic crystalloid in septic sheep was similar to control animals and septic sheep also were able to maintain similar levels of PV expansion in response to fluid infusion. The difference between the septic rabbit model (34) and septic sheep model (40) was postulated to be due to the different hypodynamic and hyperdynamic septic shock states that may have been encountered by the models.

Following these studies, fluid dynamics were explored subsequent to administration of vasoactive agents (dopamine, isoprenaline, phenylephrine) in a population of healthy awake sheep (43). Vasoactive drugs were discovered to markedly change the distribution and elimination of crystalloid fluid, thereby altering the PV expansion, urinary excretion, and the risk of peripheral edema (43). An α_1_-adrenergic receptor agonist (phenylephrine) was found to promote renal excretion of infused fluid at the expense of fluid distribution to the periphery thus limiting the volume expanding effects of the fluid infusion, while β_1_-adrenergic receptor agonist (isoprenaline) had the opposite effect. The interaction between fluid administration and vasoactive drug (norephinephrine, phenylephrine, dopamine, and esmolol) delivery was recently further explored in an experimental sepsis model in sheep (45). Results were similar in most respects to the healthy sheep model (43) in that α_1_-adrenergic receptor stimulation with vasoactive drugs accelerated the distribution and elimination of infused fluid, while β_1_-adrenergic receptor stimulation retarded the distribution and elimination of infused fluid. Having said that, α_1_-adrenergic receptor stimulation did not increase urinary excretion (elimination) to the same extent as it did in healthy awake sheep (43) due to the compounding inhibitory effects of sepsis, general anesthesia, and hypovolemia on diuresis, therefore worsening overall peripheral fluid accumulation (45). In addition, the tendency for peripheral fluid accumulation in the septic sheep model was also pronounced due to the virtual absence of fluid redistribution from peripheral tissues back to the central compartment, in particular when phenylephrine was given (45). The results of this study suggest that crystalloid fluids given in the early phase of sepsis have a marked tendency to accumulate in extravascular peripheral tissues, and drugs that exert a strong stimulating effect on β_1_-adrenergic receptors help to limit this aberrant fluid distribution thereby improving hemodynamics through more effective PV expansion.

Aside from the aforementioned animal research models performed by the same research group, there is only one veterinary study that investigated fluid dynamics of various IV fluid solutions in a prospective crossover experimental study involving 4 healthy anesthetized dogs (range 23–25 kg) (20). Silverstein and colleagues utilized a non-invasive continuous in-line hematocrit monitor (Crit-Line^TM^ IIR hematocrit monitor, In-Line Diagnostics, Kaysville, UT) that optically measured hematocrit along an extracorporeal circuit established between a central venous jugular catheter and a cephalic venous catheter to describe the real-time BV changes over 4 h, following rapid IV fluid administration (20). This monitor provides hematocrit value and calculates percent change in BV every 20 s. Each dog served as its own control and received the following treatments on separate occasions every week: 80 mL/kg of 0.9% NaCl at 150 mL/min, 20 mL/kg of Dextran 70 at 150 mL/min, 30 mL/kg of 6% hetastarch at 150 mL/min, 4 mL/kg of 7.5% NaCl at 1 mL/kg/min, and no IV fluid as control (20). In this study, immediate and rapid increases in BV were described during the infusion of each IV fluid bolus. The volume expansion effect of each IV fluid bolus was directly influenced by the volume of fluid administered, with 0.9% NaCl resulting in the greatest increase in BV (76.4 ± 10.0% change) followed by synthetic colloids (35.9 ± 7.3% change for Dextran 70; 27.2 ± 6.4% change for HES), and finally 7.5% NaCl (17.1 ± 3.2% change) immediately at the end of the infusions (20). Although the volume expanding effect of HS was significantly less than that of any other fluid, its efficiency ratio was the greatest (2.7 ± 0.5) of all the fluids while the efficiency ratio for 0.9% NaCl was the smallest (0.8 ± 0.1). The efficiency ratio for Dextran 70 and HES were 0.9 ± 0.4 and 1.1 ± 0.3 respectively. Upon discontinuing both 0.9% NaCl and HS infusions, the rise in BV ceased immediately with a steep decline in BV for 10 min followed by a more gradual decline thereafter. The fall in BV in the 0.9% NaCl group fell below that of HES by 30 min post-infusion. In contrast, the rise in BV continued for 10 min at the end of colloid infusions and a plateau was observed for the remainder of the experiment. By 240 min, colloidal solutions sustained the greatest volume expansion (25.6 ± 16.1 and 26.6 ± 8.6% for Dextran 70 and HES, respectively) compared to the crystalloid solutions (18.0 ± 9.7 and 2.9 ± 6.1% for 0.9% NaCl and HS, respectively) (20). Similar to VK studies of healthy conscious human volunteers (32), isotonic crystalloid resulted in a substantial BV increase during the infusion period (76.4 ± 10.0% change) and distributed into the ECF compartment by 30 min post-infusion with only ~25% of the delivered volume remaining within the intravascular space (20).

Following publication of this study by Silverstein and colleagues, IV fluid expansion and retention of differing resuscitative fluids have been widely disseminated in the veterinary literature and textbooks and generalized to the feline species. The use of a non-invasive continuous in-line hematocrit monitor in Silverstein's study was an innovative and unique method which obviated the need for repeated invasive blood sampling, the use of sophisticated mathematical modeling, or isotope administration to estimate BV. However, this method requires placement of a central venous catheter, heavy sedation or general anesthesia, and the use of an extracorporeal circuit which is not practical in the clinical setting, except for patients receiving renal replacement therapies.

## Limitations of Volume Kinetics

As mentioned previously, VK has not found widespread adoption in human medicine due to the complexity of these analyses requiring some mathematical and statistical background, combined with an understanding of biology, pharmacology, and physiology. Volume kinetic modeling and analysis involves specialized PK modeling software that utilizes distinctive PK nomenclature and programming languages which may not be intuitive to the general scientific audience. Data collection is simple and straightforward; however, data analysis and interpretation involve a steep learning curve for those who are unfamiliar with these concepts. Training courses and first-hand utilization of the PK modeling software would be able to accelerate this learning curve.

The main restriction to extensive clinical use of VK is the requirement for repetitive invasive Hb sampling (134). Successive blood sampling over a period of time, usually 3–4 h, is necessary to capture and understand the dynamic fluid flux within the body (32). This presents a greater challenge in veterinary patients who are much smaller with unforgiving blood volumes. A non-invasive modality that could continuously trend a patient's Hb level with accuracy and precision is essential for the safe translation of VK investigation to the clinical population. A continuous non-invasive Hb monitoring device (Radical-7® Pulse CO-Oximeter®, SET V7.4.0.9 and SET V.6.0.1, Handheld R.7.7.1.0, D-station R5.1.2.7, Masimo Corp., Irvine, CA, USA) successfully generated plasma dilution curves and useful kinetic data for group-level human VK analyses, but was unsuccessful in replacing invasive blood sampling due to wide between-subject variation and exaggeration of plasma dilution (134–136). This technology, in terms of hardware and proprietary software algorithms, has been continuously improved over the years; thus, it is worthwhile to reexplore its potential in VK analysis. Unfortunately, this non-invasive Hb monitoring technology is not ready for veterinary use following unsuccessful validation in anesthetized dogs (137) and the lack of published studies in cats.

Mass balance technique using laboratory blood Hb changes has been widely used to estimate intravascular volume status (21, 138–140) but its accuracy has recently been criticized (92, 141) despite past validation against the radio-iodinated serum albumin external indicator-dilution technique (21). Given the possible ripple effect of measurement errors on volume estimation (92), collection of duplicate or triplicate Hb samples, adequate mixing of sampled blood (142), and high-precision laboratory analysis are important to reduce between-sample variability that would affect serial plasma dilution determination and subsequent VK analysis (32).

Recommendations from published VK studies include utilizing a safe yet adequate IV infusion volume, e.g., 20–25 mL/kg crystalloid fluid bolus over 30 min, to minimize “noisy” data and estimation errors associated with very small infusion volumes (32, 92). Many fluid dynamic studies have infused fluids over 30 min to several hours (17, 143) and these prolonged infusion times preclude the differentiation of actual volume effects from distribution-excretion effects (20). Lastly, physiologic variations as a result of a change in body position, splenic contraction and sequestration, dehydration, hypotension, and the use of diuretic or adrenergic drugs, can affect extrapolation of RBC mass to vascular volume changes (20, 32) and should be minimized during the study period. Although of little importance in humans (144), catecholamine-mediated splenic contraction is known to cause a transient increase in hematocrit and BV in dogs, cats, and sheep (31, 145–148) making splenectomy an important consideration in the experimental research setting.

## Conclusions

Volume kinetics is an innovative research method that is gaining recognition for its wealth of accumulated evidence in this new era where clinicians are searching for context-sensitive fluid therapy paradigms. Despite its limitations, the pharmacokinetics of IV fluid therapy is still an appealing concept that has the potential to serve as a new research tool in veterinary medicine to provide insights on the distribution and elimination of commonly prescribed IV fluids. This research method is feasible and deserves a thorough investigation in the companion animal species. The detailed time course of IV fluids within the body, volume expansion effect, efficacy, half-life (duration of effect), and body water physiology in different patient populations under various clinical conditions may allow for more evidence-based IV fluid therapy prescriptions for our veterinary patients. Until a less invasive method of serial Hb monitoring has been validated for veterinary patients, VK will likely remain as a fundamental research tool for modeling and simulation of IV fluid therapy.

## Author Contributions

XY wrote the first draft of the manuscript. SB, AB, WM, and RH provided critical revision of the manuscript. All authors read and approved the final version of the manuscript to be published.

## Conflict of Interest

The authors declare that the manuscript was written in the absence of any commercial or financial relationships that could be construed as a potential conflict of interest.

## Nomenclature

**Table:**

IV	Intravenous
PK	Pharmacokinetics
VK	Volume kinetics
1-VOFS	One-volume fluid space
2-VOFS	Two-volume fluid space
Hb	Hemoglobin (g/dL)
PV	Plasma volume
BV	Blood volume
*V*_*d*_	Volume of distribution
*V*	Volume of expandable body fluid space (mL)
*v*	Volume of expanded body fluid space (mL)
*v* − *V*	Absolute volume expansion for 1-VOFS model (mL)
*v*_*c*_ − *V*_*c*_	Absolute volume expansion for 2-VOFS model (mL)
$\frac{\left(v-V\right)}{V}$	Fractional volume expansion or plasma dilution for 1-VOFS model (mL)
$\frac{\left(v_{c}-V_{c}\right)}{V_{c}}$	Fractional volume expansion or plasma dilution for 2-VOFS model (mL)
*V*_*c*_ = *V*_1_	Volume of expandable central body fluid space (mL)
*v*_*c*_ = *v*_1_	Volume of expanded central body fluid space (mL)
*V*_*d*_	Volume of distribution (mL)
*V*_*p*_ = *V*_*t*_ = *V*_2_	Volume of expandable peripheral body fluid space (mL)
*v*_*p*_ = *v*_*t*_ = *v*_2_	Volume of expanded peripheral body fluid space (mL)
*Cl*	First-order clearance (mL/min)
*Cl*_*R*_	Renal clearance (mL/min)
*Cl*_*d*_	Distribution clearance (mL/min)
*Cl*_0_	Zero-order clearance (mL/min)
*k*_10_	First-order elimination rate constant (/min)
*k*_12_	Central to peripheral intercompartmental rate constant (/min)
*k*_21_	Peripheral to central intercompartmental rate constant (/min)
*k*_0_ = *k*_*b*_	Zero-order elimination rate constant (/min)
*R*_0_ = *k*_*i*_	Intravenous infusion rate (mL/min)
AUC	Area under the concentration or plasma dilution-time curve
*t*_1/2_	Elimination half-life (min)
BW	Body weight
*f*_*t*_	Translocated volume (mL)
HES	Hydroxyethyl starch
HS	Hypertonic saline
HSD	Hypertonic saline-dextran
*MHb*	Total hemoglobin mass (g/dL)
NaCl	Sodium chloride
RBC	Red blood cell(s)
